# Supplementary material for: Preclinical Evaluation of a Novel Series of Polyfluorinated Thalidomide Analogs in Drug-Resistant Multiple Myeloma
Source: Biomolecules. 2024 Jun 19;14(6):725. doi: 10.3390/biom14060725 (PMC11201495; doi:10.3390/biom14060725)
Supplement: Supplementary file 1 [file biomolecules-14-00725-s001.zip › biomolecules-3026091-supplementary.pdf]

Supplementary Figure

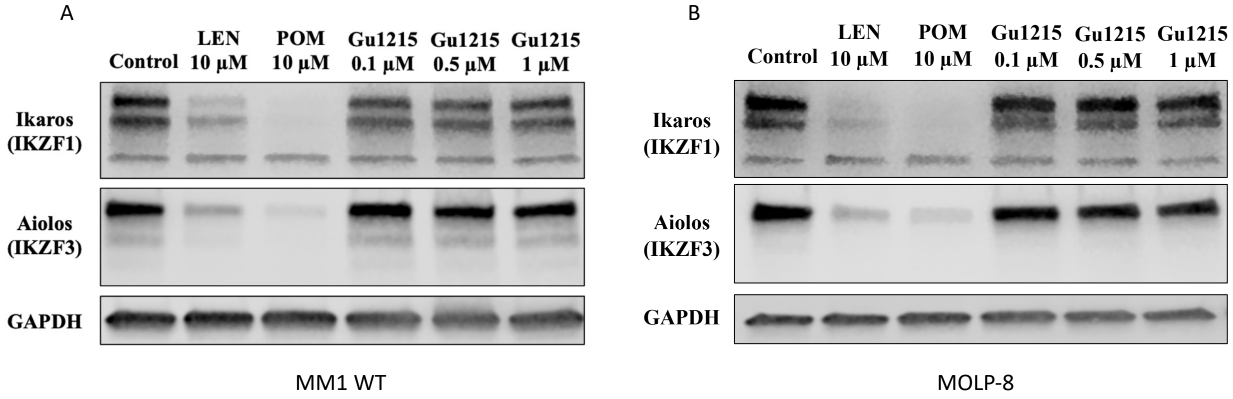

Supplementary Figure S1. Western blot analysis of cereblon neosubstrates in myeloma cell lines. Expression of Ikaros and Aiolos proteins remain unaffected after 24-hour treatment of both MM1.S (A) and MOLP-8 cells (B) with Gu1215 at 0.1 μM, 0.5 μM, and 1 μM. Results shown are representative of at least three independent experiments.
